# Measurement of Water Saturation in Soybean Oil

**DOI:** 10.1021/acsomega.3c00348

**Published:** 2023-05-23

**Authors:** Bat-Sheva Galmidi, Mark A. Iron, Naomi Zurgil, Mordechai Deutsch

**Affiliations:** † The Biophysical Interdisciplinary Jerome Schottenstein Center for the Research and Technology of the Cellome, Physics Department, Bar Ilan University, Ramat-Gan 5290002 Israel; ‡ Computational Chemistry Unit, Department of Chemical Research Support, Weizmann Institute of Science, Rehovot 7610001 Israel

## Abstract

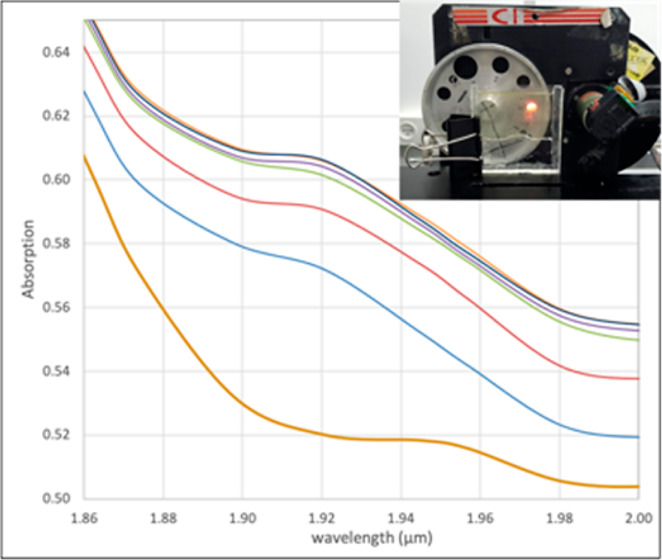

In a previous study, it was observed that survivability was low when attempting to cryopreserve sperm cells in a nanoliter-sized droplet protected under soybean oil, in stark contrast to the high survival rates in milliliter-sized droplets. In this study, infrared spectroscopy was used to provide an estimate of the saturation concentration of water in soybean oil. By following the time evolution of the infrared absorption spectrum of water–oil mixtures, the saturation of water in soybean oil was found to reach equilibrium after 1 h. From the absorption spectra of neat water and neat soybean oil and the application of the Beer–Lambert law to an estimation of the absorption of a mixture from its individual components, it was estimated that the saturation concentration of water is 0.010 M. This estimate was supported by molecular modeling using the latest semiempirical methods (in particular, GFN2-xTB). While for most applications the very low solubility has little impact, the implications in those exceptions were discussed.

## Introduction

Cryopreservation of individual preselected sperm cells is a well-recognized necessity,^1^ as exemplified by some recent clinical studies.^2−5^ While attempting to freeze sperm cells inside nanoliter droplets protected under oil, a worrying phenomenon was discovered: the cells did not survive the freeze–thaw cycle. This is in contrast to the survivability in larger (microliter) droplets, which generally yield high survival rates similar to control experiments (freezing in macrotubes with a volume of ∼1 mL). It was found that the low survival rates in nanoliter droplets is due to diffusion of water from the pre-freezing droplet into the covering oil medium. This elevates the solute concentration within the droplet, thereby injuring the enclosed cells. Unfortunately, the rate of water diffusion sharply increases for droplets smaller than a few nanoliters, whence sperm cell death is observed.^6^ In related studies, the water content of oils was found to have an impact on the heat resistance of bacterial spores^7,8^ and to reduce the efficacy of machine oil in protecting metal parts.^9^ An analytical analysis of these findings indicates that saturation of the oil environment with water significantly lowers the mortality rate after thawing, even in volumes smaller than 1 nL. We used an infrared spectrometer to measure the absorption spectrum of the oil, and the spectra were compared to those of the oil mixed with water. In the study presented here, we will provide an estimate for the saturation concentration of water in soybean oil, a cheap oil often used to protect the droplets during cryopreservation.

## Materials and Methods

Soybean oil was purchased from the local grocery store.

Measurements were performed using a CI-Systems SR5000N spectrometer with a configuration of a silicon/InGaAs detector and CVF7 (for the spectral band, 0.4–2.4 μm) and a narrow FOV of 7 mrad. The radiation source was a CI-Systems SR200 cavity blackbody set to 1000 °C.

All experiments are carried out in a temperature-controlled clean room with the room thermostat set to 25 °C.

We used an optical chopper mounted on the blackbody radiation source and a lock-in-amplifier on the radiometer side to ensure that the only signals measured are those emitted from the source and not radiation that was reflected or emitted from ambient sources nor the self-emission of the samples. Each spectrum is an average of 10 spectral scans.

**Figure 1:**
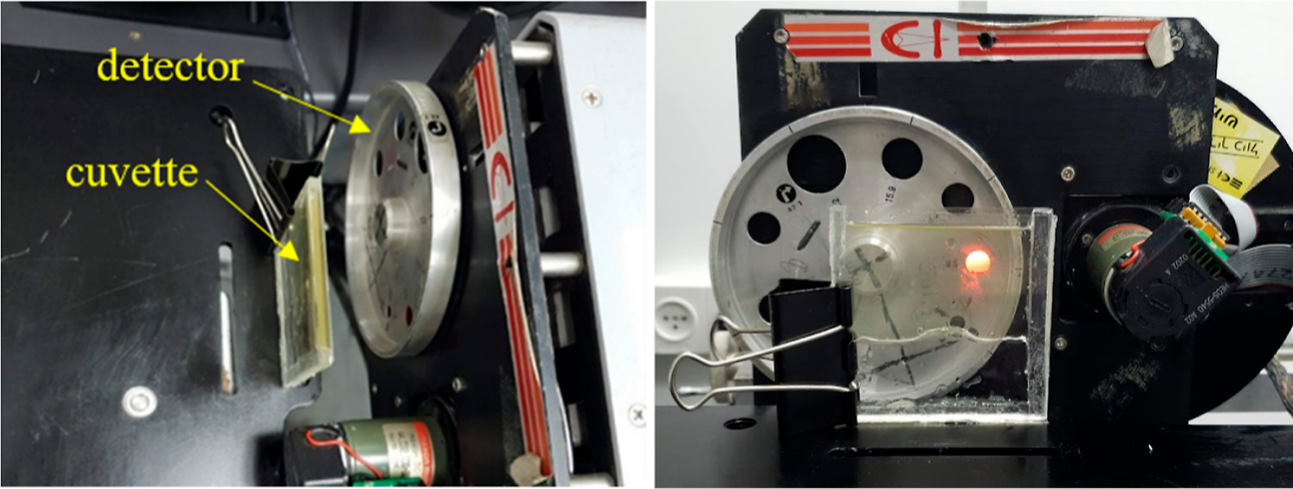
Above (left) and front (right) views of the cuvette and the detector. (Photographs by Bat-Sheva Galmidi, Bar-Ilan University).

Cuvettes were made from two parallel 75 × 75 × 1.2 mm^3^ microscope glass slides connected by a 5 mm thick sealing layer of Billicon silicone tape. While one would intuitively assume that an accurate measure of the cuvette thickness would be essential to the study, given the dependence of the Beer–Lambert law (eq 5) on the optical path length (*l*), this quantity actually cancels out in the derivations of eqs 4 and 8 (vide infra). Our experimental setup is shown in Figure 1.

The intensity of the transmitted light (S) was measured twice at each wavelength: once with an empty curvet (*S*_0_) and again filled with the sample (*S*). *S*_0_ and *S* are the spectral irradiance at optical path length *l*. Since the signal strength is linearly proportional to the power, the following equations are used to describe the measured signal^10^
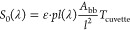
1
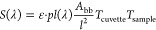
2where ε is the emissivity of the blackbody, *pl*(λ)is the Planck function at the blackbody temperature in units of spectral radiance , *A*_bb_ is the solid angle subtended by the blackbody using its area and distance (this converts the radiance to irradiance), *T*_cuvette_is the spectral transmission of an empty cuvette, and *T*_sample_ is the spectral transmission of the sample.

*T*_sample_ was found by dividing eq 2 by eq 1, which gives
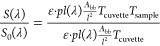
3Because both measurements were conducted with exactly the same system (cuvette, blackbody radiation source, temperature, size, and distance), *T*_cuvette_ and the system parameters  cancel out leaving
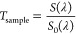
4Due to the optical chopper, it can be assumed that reflection is negligible. Thus, since the sum of absorption (*A*), reflection (*R*), and transmission (*T*) is 1, then the absorption of the sample is *A* = 1 – *T*_sample_.

## Computational Methods

Solvation free energies were determined using Grimme and co-workers’ Crest version 2.12.^11,12^ In particular, the quantum cluster growth (QCG) module^13^ was used in conjunction with their GFN2-xTB semiempirical (tight-binding based) method^14^ and associated xTB program (version 6.4.0);^15^ GFN2-xTB was specifically parameterized for geometries, frequencies, and noncovalent interactions (hence its acronym) and thus should be applicable to our problem. Solvation was modeled using the analytical linearized Poisson– Boltzmann (ALPB) implicit solvation model^16^ with either water or benzene (vide infra). CREST (an abbreviation of conformer–rotamer ensemble sampling tool) is a tool to sample to conformer space of a system and interfaces with their xTB program that provides energies. The QCG module adds solvent molecules around a solute, primarily by screening docking positions, and calculates the solvation free energies explicitly. In this study, the solute was a single water molecule (vide supra) solvated in water, oleic acid, or linoleic acid. Ensembles of 10–40 solvent molecules were considered with either four or six conformers used to calculate the solvation free energies. To further model reality, the clusters were embedded in an implicit solvent continuum; since oleic (ε = 2.336^17^) and linoleic (ε = 2.754^17^) acids are not available solvent in the model, benzene (ε = 2.2825^17^) was used as this solvent has the closest dielectric constant (ε) of the solvents available in xTB. To give a statistical estimate of the uncertainty in the calculation, each solvation-free energy calculation was repeated five times for each ensemble; it should be noted that for the lipids, many of the calculations failed to run to completion and a few gave clearly unphysical results. The results of the individual Crest runs that ran to completion are given in the Supporting Information in Table S2.

## Results and Discussion

**Figure 2:**
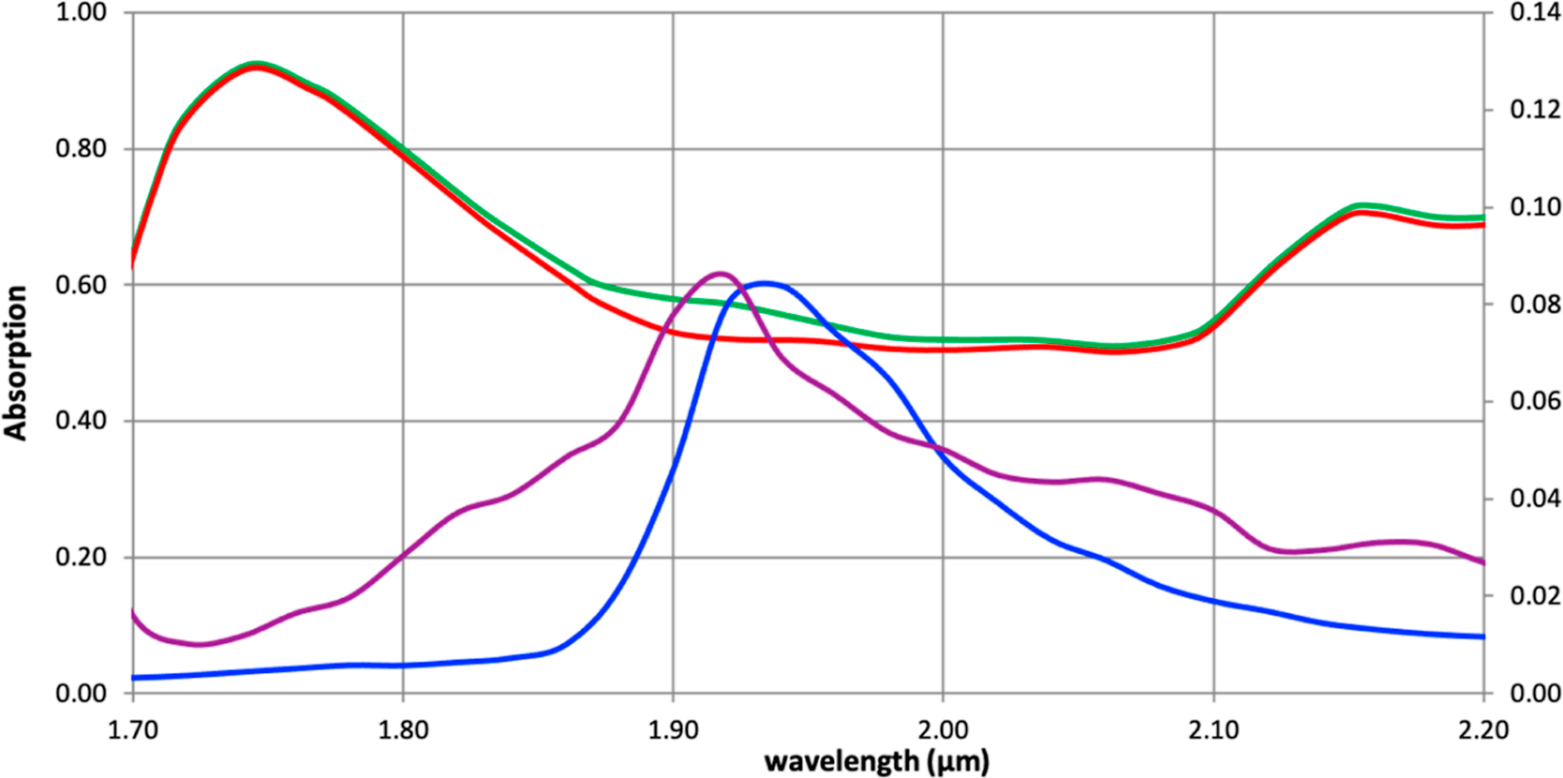
Infrared absorption spectra of water (blue, left axis, scaled by 1/200 to improve visibility), dry oil (red, left axis), and saturated oil (green, left axis) in the region of the primary absorption peak of water. Also shown in the difference spectrum (purple, right axis) between saturated and dry oil.

The absorption spectra of three samples are shown in Figure 2: double-distilled water (DDW), soybean oil (hereinafter for simplicity just “oil”), and soybean oil saturated with water (hereinafter “saturated oil”). The saturated oil mixture was prepared by layering equal amounts of oil over water, letting it sit for 12 h, and decanting the oil layer into the cuvette.

The absorption spectrum of oil has two main features, at 1.743 and 2.16 μm, while water has an absorption at 1.94 μm. The spectrum of the saturated oil for the most part mirrors the spectrum of the dry oil sample. There is, however, a noticeable deviation around the absorption of water, which becomes more apparent if one subtracts the spectrum of the saturated oil from that of dry oil (Figure 2, red line). The resulting difference spectrum resembles the absorption spectrum of water but is blue-shifted by 0.02 μm. This shift is likely due to the oil affecting the hydrogen-bonding network of water.

### Saturation Concentration of Water in Oil

**Figure 3:**
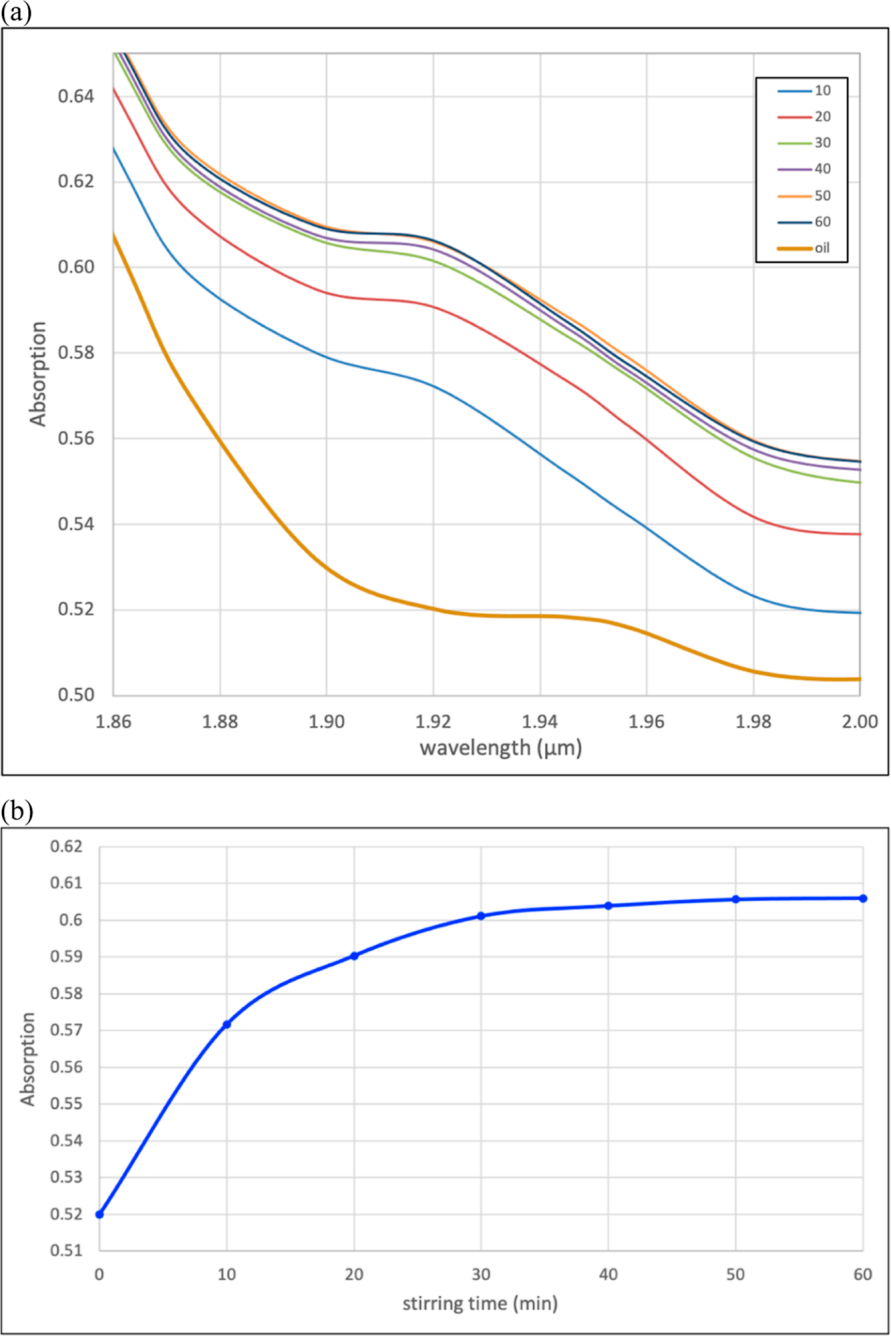
Time evolution of the IR absorption spectrum of a stirred 4:1 soybean oil/water mixture. (a) Spectrum in the region of the main water feature at different stirring times. (b) Asymptotic behavior of the absorption at 1.921 μm with stirring time.

To understand the effects of water solubility in oil on our cryopreserved samples, the saturation concentration of water in soybean oil needed to be determined. Soybean oil and DDW (minimum 4:1 ratio) were mixed with a magnetic stirrer bar in a glass vial at 25 °C. A low stirring speed (30 rpm) was used to avoid bubbles that would cause light reflections. The absorption spectrum was measured every 10 min for 60 min (Figure 3a) by transferring by pipette the oil layer from the vial to the cuvette. After 50 min, the oil reached saturation, and continued stirring did not increase water concentration; this is further emphasized by the asymptotic behavior of the absorption at 1.902 μm, the center of the primary water absorption (Figure 3b). It should be noted that these results refer to the specific geometries of the glass vial and the magnetic stirrer bar and of the stirring rate. At smaller oil/water ratios, saturation was reached faster.

### Estimation of Water Concentration in Saturated Oil

We used the Beer–Lambert law to assess the saturation concentration of water dissolved in oil. The Beer–Lambert law describes the absorption of light passing through an optical path *l* through a medium (solvent) with an absorbing species (solute) with a molar absorption coefficient ε(λ) and concertation *C*
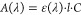
5The optical path *l* is a function of the geometry of the sample (in our case, the thickness of the cuvette or 0.5 cm). The concentration of a pure liquid can be determined from its density (ρ) and molecular weight (M_W_) according to  (a factor of 1000 may be needed to balance the units). Thus, ε(λ) can be extrapolated by measuring the absorption. Patzek reported, based on a report by Perkins,^18^ an average molecular weight for soybean oil, based on the weighted average of the fatty acids that comprise it (primarily 52.0% linoleic acid, 25.0% oleic acid, and 12.0% palmitic acid) of *M*_W_^oil^ = 920 g/mol; the reported average density is 0.9138 g/mL.^19^ Thus, the concentration of soybean oil is 0.993 M. It should be noted that these are average values for soybean oil as the oil’s composition and density will vary somewhat based on the source of the soybeans and extraction of the oil from the raw material;^18^ these nuances, however, are beyond the scope of this study. Likewise, the concentration of water is 55.56 M.

When there are multiple absorbers in the same solution, the absorption will be a linear combination of their individual absorbances
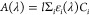
6In our case
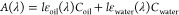
7The molar absorption coefficients for oil and water are taken from the absorptions of the neat samples. Substituting eq 5 for each solvent into eq 7, one obtains
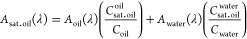
8Thus, one has two evaluations of the absorption of water-saturated oil: direct measurement and using the Beer–Lambert law. The latter has two unknowns—the concentrations of water and oil in the saturated oil (*C*_sat.oil_^water^ and *C*_sat.oil_^oil^)—and these can be found by minimizing the mean absolute deviation (MAD) between the two; this was accomplished using the “Solver” function of Microsoft Excel for each measured wavelength between 1.7 and 2.2 μm (the data is provided in the Supporting Information in Table S1). This gives estimations for the concentrations of *C*_sat.oil_^water^ = 0.010 M and *C*_sat.oil_^oil^ = 1.040 M.

### Computational Estimate of the Saturation Concentration of Water in Soybean Oil

Da Silva et al. derived an equation for the transfer free energy (TFE) of a solute from solvent *i* to solvent *j*(20)
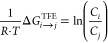
9where *C*_*i*_ is the concentration of the solute in solvent *i*. For our purposes, we shall consider the transfer of a water solute from water to either oleic or linoleic acid. The fact that soybean oil is actually a mixture of many components, even though there are only two primary components, is a complication beyond the scope of this project. It should be noted that Da Silva’s derivation assumed low solute solubility—which is problematic for water dissolved in water—allowing them to consider the solute as infinitely dilute; nevertheless, we will assume that the consequences of violating this assumption are smaller than the consequences of the other approximations.

By definition, the TFE is the energy required to transfer the solute from solvent *i* to solvent *j*, which is thermodynamically equivalent to transferring the solute from solvent *i* to the gas phase (i.e., the negative solvation energy or −Δ*G*_*i*_^solv^) and thence to solvent *j*, or
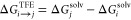
10For convenience, we choose solvent *i* to be either oleic or linoleic acid and solvent *j* to be water. Rearranging Da Silva’s equation gives
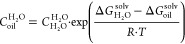
11The concentration of water in water, as noted above, is  ≈ 55.56 M, the universal gas constant *R* ≈ 0.001987 kcal/mol·K, and *T* = 25 °C.

The calculations herein only consider individually a single component of the complex mixture that forms soybean oil. Considering the entire mixture would be computationally infeasible, but one can assume that solubility varies linearly between each component; this is a rough approximation, likely small compared to the other approximations already made, yet it is reasonable to assume that it at least varies monotonically. Likewise, for simplicity, we will assume that the oils are mixtures of only the two primary components in the (normalized) ratios listed above.

All that remains is to calculate the solvation free energies of water in water and in the two fatty acids. Grimme and co-workers’ program Crest has a module that, using their fast yet reliable GFN2-xTB method, does exactly that. (See the Computational Methods section for details.) For these purposes, Crest was used to build clusters of water solute molecules surrounded by *n* solvent (water, oleic acid, or linoleic acid) molecules (*n* = 10–40), and this cluster was embedded in an implicit solvation model (either water or benzene). In each case, either four or six solute–solvent clusters were considered, and each calculation was repeated five times. This potentially gives forty results, which would allow for an estimate for the error; however, many of the Crest runs failed to run to completion for various reasons, and a few were discarded as the results were too far away from the average (beyond 2σ) to be physically meaningful (the results of each run are given in the Supporting Information in Table S2).

The results of the calculations are shown in Table 1. This gives estimations for the solubility of water in these solvents (i.e., ). From the results for the solubility of water in oleic and linoleic acids, the two primary components of soybean oil, one can estimate that the saturation concentration of water in soybean oil is 0.219 M. This is somewhat higher than measured by IR absorption spectroscopy, but one needs to consider that the various runs gave large standard deviations, especially for the fatty acids, and the exponential function means that these are magnified when considering the saturation concentrations; if one were to consider ±1σ errors, then a much lower saturation concentration is conceivable. Furthermore, the minor components of soybean oil will have some impact on the results. (It should be noted that this is only a measure of the equilibrium water concentration of water in the oil and is not a measure of the rate of solvation, which is beyond the scope of this study.)

**Table 1:** Calculated Results for the Solvation of Water in Oleic and Linoleic Acids; See the Text for Definitions of Measures

		oleic acid	linoleic acid	water	
*n*_calculations_	units	30	23	39	“soybean oil”^a^
Δ*G*_*i*_^solv^	kcal/mol	–2.51 ± 1.16	–3.20 ± 1.67	–6.10 ± 0.71	
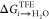	kcal/mol	–2.90	–3.58		
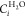	mol/L	0.4178	0.1311		0.219
min(±1σ)^b^	mol/L	0.0026	0.0075		0.004

a Calculated as a weighted average of 31% oleic acid and 69% linoleic acid, the normalized percentages of each fatty acid in soybean oil if these were the only two components (see text).

b Calculated solubility assuming the least favorable combination of ±1σ on the solvation free energies (see text).

## Implications for Cryopreservation

The impetus behind this study was the problem that sperm cells could be preserved in larger droplets but not in nanoliter-sized droplet; this was a significant technical challenge in our project in cryopreserving a single sperm cell.^6^ Oil was used to protect the droplets during the cryogenic process because water is generally considered immiscible in oils like soybean oil. It is generally understood that this is a rough approximation that in practice does not have an impact on experiments and that some amount of water, albeit very small, does dissolve. Here, we have quantified this “very small amount.” For larger water droplets, this low solubility clearly is not a problem. However, as one moves to smaller droplets, the oil/water ratio significantly increases, and given the small volume of the nanoliter droplet, relatively more water is absorbed by the soybean oil. The larger droplets clearly can survive this loss of water but the nanoliter droplets cannot, leading to loss of sperm cell survivability.

Two solutions were found for this problem. One was to use a special mineral oil, but this oil is expensive and only comes in small quantities. In this previous study, droplets of distilled water were injected under either mineral or soybean oil. These droplets were monitored with bright-field microscopy with images acquired at five-minute intervals. The shrinkage rates of droplets (measured as the change in cross-sectional area with time) were d*A*/d*t* = 88.4 and 495 μm^2^/min for mineral and soybean oil, respectively. Another solution is to saturate the soybean oil with water before using it, and this led to a reduction in the diffusion rate of the water into the oil and increased the survival rate of the cells after the freeze–thaw cycle.^6^

## Conclusions

While it is commonly accepted that oils, like soybean oil, cannot dissolve water, this misconception is put to rest here. By measuring the infrared absorption of oil–water mixtures, we were able to quantify the solubility of water in soybean oil (0.010 M). These measurements were supported by computational modeling using some of the latest semiempirical methods. A more accurate statement would be that the solubility of water in soybean oil is sufficiently low as to be negligible in most applications, but it is in those exceptions where the low solubility cannot be ignored or neglected. One such case is our cryopreservation of a single sperm cell in a nanoliter-sized droplet. By understanding that the low solubility was nevertheless problematic, we were able to overcome this obstacle, thereby opening new vistas in the field of cryopreservation in small volumes.

## Supporting Information Available

Absorption spectra of soybean–water mixtures as a function of stirring time and results of the solvation free energy calculations (PDF)
