# Supplementary material for: Measurement of Water Saturation in Soybean Oil
Source: ACS Omega. 2023 May 23;8(22):19385–90. doi: 10.1021/acsomega.3c00348 (PMC10249122; doi:10.1021/acsomega.3c00348)
Supplement: Supplementary file 1 — ao3c00348_si_001.pdf [file ao3c00348_si_001.pdf]

Measurement of Water Saturation in Soybean Oil

Bat-Sheva Galmidi,[a] *Mark A. Iron,*[b,*] *Naomi Zurgil,*[a] *and Mordechai Deutsch*[a,*]

[a] The Biophysical Interdisciplinary Jerome Schottenstein Center for the Research and Technology of the Cellome, Physics Department, Bar Ilan University, Ramat-Gan, 5290002 Israel.

[b] Computational Chemistry Unit, Department of Chemical Research Support, Weizmann Institute of Science, Rehovot, 7610001 Israel.

Supporting Information

Table S1. Absorption as a function of wavelength (λ, μm) of water, soybean oil and water-oil mixtures after 10-60 minutes stirring.

λ	water	oil	10	20	30	40	50	60
1.302	0.004036	-0.045941	-0.028198	0.036954	0.081395		0.098201	0.094101
1.321	0.005018	-0.044870	-0.028540	0.033707	0.082490	0.091496	0.097578	0.095851
1.340	0.006655	-0.004978	-0.003465	0.058364	0.102272	0.107521	0.117186	0.112078
1.361	0.010073	-0.011494	0.015663	0.073727	0.119412	0.128617	0.133471	0.135772
1.385	0.022182	0.156879	0.171332	0.216315	0.248441	0.254841	0.261903	0.257406
1.409	0.045055	0.229186	0.258407	0.299823	0.330784	0.335373	0.340999	0.338609
1.433	0.080436	0.236546	0.258583	0.300776	0.332375	0.337835	0.342175	0.341997
1.445	0.104727	0.199842	0.222266	0.267328	0.297779	0.305178	0.308913	0.308637
1.457	0.103273	0.160111	0.180475	0.229834	0.259622	0.267465	0.271495	0.270907
1.480	0.077200	0.101372	0.113274	0.164478	0.197729	0.203106	0.209008	0.206366
1.504	0.063964	0.049766	0.059857	0.109209	0.147085	0.153404	0.158532	0.157753
1.516	0.051091	0.034298	0.046028	0.095740	0.132503	0.140354	0.144646	0.145079
1.540	0.043018	0.023711	0.031744	0.081725	0.113552	0.121361	0.127219	0.124967
1.564	0.035164	0.018122	0.023009	0.071158	0.105004	0.109745	0.116882	0.113498
1.588	0.028909	0.012018	0.019537	0.067139	0.103540	0.109814	0.114742	0.114762
1.600	0.024436	0.014715	0.020781	0.067622	0.102112	0.109826	0.114205	0.114583
1.623	0.021164	0.043005	0.045749	0.088659	0.116919	0.123619	0.129925	0.126710
1.647	0.018109	0.129350	0.133371	0.170855	0.198887	0.202192	0.208752	0.205290
1.659	0.016509	0.199228	0.203890	0.236424	0.264167	0.267231	0.272645	0.270389
1.683	0.016327	0.428530	0.432938	0.450175	0.468164	0.471492	0.475287	0.472980
1.707	0.016145	0.727636	0.733674	0.736321	0.743152	0.743698	0.746692	0.743550
1.719	0.018582	0.839636	0.846981	0.846281	0.850493	0.850371	0.852163	0.849897
1.743	0.022327	0.917424	0.923502	0.925791	0.928362	0.928853	0.929257	0.929238
1.766	0.025964	0.885408	0.893244	0.897455	0.900352	0.901211	0.901569	0.901952
1.778	0.029527	0.856923	0.866330	0.871599	0.874974	0.875928	0.876674	0.876722
1.802	0.029200	0.781973	0.793338	0.802408	0.808334	0.809356	0.810445	0.810448
1.826	0.032655	0.704645	0.717425	0.729600	0.738243	0.739844	0.741247	0.741849
1.838	0.037236	0.668691	0.683828	0.696056	0.705247	0.707443	0.709114	0.709595
1.862	0.051600	0.601764	0.622847	0.636952	0.646104	0.648206	0.651076	0.650353
1.874	0.113018	0.570624	0.598584	0.613269	0.623134	0.624533	0.627514	0.626446
1.898	0.240509	0.531752	0.579966	0.594804	0.606534	0.607643	0.610283	0.609799
1.921	0.416509	0.519929	0.571614	0.590285	0.601063	0.603830	0.605592	0.605941
1.945	0.435745	0.518284	0.551983	0.573413	0.583961	0.585919	0.588563	0.587401
1.957	0.384691	0.515723	0.541614	0.562509	0.574219	0.575525	0.578500	0.577041
1.981	0.334655	0.505330	0.522684	0.541112	0.554960	0.556974	0.559067	0.558908

λ	water	oil	10	20	30	40	50	60
2.005	0.251345	0.504017	0.519011	0.537473	0.549138	0.552262	0.554342	0.554154
2.029	0.203600	0.507978	0.519418	0.538695	0.549652	0.551468	0.554462	0.552954
2.041	0.163527	0.507851	0.517107	0.536278	0.548418	0.549696	0.552781	0.551358
2.065	0.141964	0.500887	0.509740	0.526975	0.540490	0.542508	0.544932	0.544896
2.088	0.114255	0.512680	0.522992	0.538515	0.549029	0.552011	0.554537	0.553776
2.100	0.097927	0.537499	0.547075	0.562270	0.571466	0.573599	0.576630	0.575082
2.124	0.087309	0.628490	0.636938	0.648149	0.656916	0.657330	0.660043	0.658385
2.148	0.074727	0.697902	0.707803	0.716971	0.724741	0.726043	0.727430	0.727424
2.160	0.067709	0.704055	0.715628	0.725151	0.731834	0.733695	0.734867	0.735100
2.184	0.062873	0.686674	0.698820	0.709071	0.715036	0.716385	0.718250	0.717417
2.208	0.060036	0.692518	0.703131	0.711015	0.717887	0.718452	0.720425	0.719451
2.220	0.058655	0.720313	0.731256	0.736299	0.742929	0.743458	0.745021	0.744502
2.243	0.060182	0.822498	0.831667	0.832390	0.835645	0.836084	0.837249	0.836541
2.267	0.062691	0.931486	0.936081	0.935026	0.936103	0.935857	0.936612	0.935909
2.279	0.072145	0.963748	0.966524	0.965524	0.966217	0.966071	0.966382	0.965902
2.303	0.081455	0.991920	0.992583	0.992426	0.992600	0.992541	0.992632	0.992623
2.327	0.097491	0.997099	0.997803	0.997317	0.997441	0.997453	0.997504	0.997447
2.339	0.116182	0.997800	0.998282	0.998029	0.998073	0.998217	0.998081	0.998093
2.363	0.135527	0.998413	0.998055	0.998595	0.998622	0.998626	0.998683	0.998786
2.386	0.157455	0.998692	0.998676	0.998847	0.998826	0.998905	0.998958	0.998974
2.398	0.182036	0.998812	0.998728	0.998944	0.999012	0.999030	0.999083	0.999070

Table S2. Results of each CREST run that ran to completion used to determine solvation free energies giving for each solvent the number of solvent molecules (n_{solv}), the number of clusters used to determine the solvation free energies ($n_{clusters}$), the run number, and the resulting solvation free energy (kcal/mol at 25 °C).

n_{solv}	$n_{clusters}$	Run Number	ΔG_i^{solv}
		**linoleic acid**	
25	4	2	-1.48
25	6	4	-1.63
25	6	5	-0.54
30	4	4	-1.98

n_{solv}	$n_{clusters}$	Run Number	ΔG_i^{solv}
		**oleic acid**	
25	4	1	-5.00
25	4	2	-5.72
25	4	4	-5.69
25	4	1	-2.51
25	6	2	-2.06
25	6	3	-1.92
25	6	4	0.08
30	4	2	-0.09
30	6	2	-2.09
		**water**	
25	4	1	-5.55
25	4	2	-5.80
25	4	3	-4.81
25	4	4	-5.29
25	4	5	-5.77
25	6	1	-5.39
25	6	2	-6.14
25	6	3	-6.17
25	6	4	-7.24
25	6	5	-6.83
30	4	1	-6.25
30	4	2	-7.05
30	4	3	-7.24
30	4	4	-6.41
30	4	5	-6.32
30	6	1	-7.04
30	6	2	-5.84
30	6	3	-5.22
30	6	4	-6.00
30	6	5	-4.86
35	4	1	-4.98
35	4	2	-6.28
35	4	3	-6.31

n_{solv}	$n_{clusters}$	Run Number	ΔG_i^{solv}
35	4	4	-6.93
35	4	5	-5.62
35	6	1	-7.04
35	6	2	-5.39
35	6	3	-6.70
35	6	4	-4.85
35	6	5	-5.85
40	4	1	-6.43
40	4	2	-5.97
40	4	3	-5.64
40	4	4	-6.08
40	4	5	-6.05
40	6	1	-6.81
40	6	2	-8.49
40	6	3	-7.18
40	6	4	-6.83
40	6	5	-5.57
**oleic acid (rejected unphysical results)**			
30	4	3	4.37
30	6	2	—[a]
**linoleic acid (rejected unphysical results)**			
30	6	5	5.94
30	4	1	-10.79
30	4	3	8243.04
30	6	1	-6516.77

[a] Value beyond print capabilities of CREST.
